# Anchorless Onlay Dynamic Anterior Stabilization of the Shoulder Using a Guided Posterior Double Endobutton Fixation

**DOI:** 10.1016/j.eats.2023.10.007

**Published:** 2024-01-01

**Authors:** Carlos Maia Dias, Rita Alçada, Manuel Ribeiro da Silva

**Affiliations:** a Hospital CUF Tejo, Lisbon, Portugal; b Hospital CUF Santarém, Santarém, Portugal; c UCMA Fidelidade, Lisboa, Portugal; d Hospital de Cascais, Cascais, Portugal; e Hospital CUF Porto, Portugal

## Abstract

The treatment of shoulder instability in the presence of a subcritical glenoid defect poses challenges, as simple Bankart seems insufficient, and the Latarjet procedure may be excessive. Recently, a dynamic anterior stabilization technique involving anterior transposition of the long head of the biceps (LHB) through a subscapularis split was described for that purpose. Previously published results demonstrated good short-term results, but several technical pitfalls have also been mentioned. We describe an onlay, anchorless, and intra-articular knotless method of fixing the LHB into the anterior glenoid that provides the important stabilizing “sling effect” of the dynamic anterior stabilization while avoiding some of the pitfalls described by other techniques.

Several surgical options have been proposed for the treatment of anterior glenohumeral instability according to age, sex, and bone loss dimension and its location. While clinical studies of Bankart repair show low complication rates, high redislocation rates have been reported even in the presence of 13% to 17% glenoid bone loss (GBL).^1^^,^^2^ These GBL values are inferior to the 20% to 25% critical values for which the Latarjet procedure has been classically proposed.^2^^,^^3^ Studies have shown that in the presence of an engaging Hill-Sachs defect, the addition of a remplissage to the Bankart repair produces better outcomes than either the Bankart repair or the Latarjet procedure alone.^4^^,^^5^ However, in cases of isolated subcritical GBL, the best option is controversial, because the Bankart repair is insufficient, remplissage does not address the GBL, and the Latarjet has a high risk of serious morbidity.^4^, ^5^, ^6^, ^7^, ^8^, ^9^, ^10^

In this context, Tang and Zhao^11^ described the transfer of the long head of the biceps (LHB) for anterior shoulder instability, immediately followed by Collin and Lädermann,^12^ who coined the procedure as dynamic anterior shoulder stabilization (DAS), a technique combining a Bankart repair with the additional sling effect of the LHB. In this procedure, the LHB was placed in a bony tunnel at the anterior glenoid margin after passing it through a subscapularis split, fixing the LHB using either a miniplate^11^ or an interference screw.^12^

In 2019, Milenin and Toussaint^13^ described an onlay technique that added the labroplasty effect to the biceps transfer to the anterior glenoid, and other authors modified the technique by using all suture anchors for that purpose,^14^ while some even performed an Hill-Sachs remplissage in addition to the DAS while not re-creating the labroplasty effect.^15^

We present a modified onlay, knotless, and anchorless DAS technique that aims to remove some of the pitfalls of the previous techniques. This work has been carried out in accordance with the Code of Ethics of the World Medical Association (Declaration of Helsinki) for experiments involving humans. All patients involved in the development of this technique were informed of the experimental nature of the procedure and gave appropriate informed consent for the procedure.

## Surgical Technique

### Patient Evaluation

A complete history is collected that must include medical history, number of previous dislocations and their etiology (traumatic vs atraumatic), medication, and allergies.

The physical examination should comprise a bilateral shoulder range of motion, both passive and active, also focusing on the scapula and the scapula-thoracic rhythm. Strength evaluation is also performed using a dynamometer, and instability tests should also be performed, including the apprehension and relocation test, load and shift and jerk tests, Gagey sign, sulcus sign, and hypermobility testing using Beighton criteria.^16^

### Imaging

Like all cases of instability, magnetic resonance imaging (MRI) and a computed tomography (CT) scan should be requested to evaluate both soft tissues (best evaluated using MRI) and bone quality (best assessed using a CT scan). The authors recommend that all sagittal and coronal cuts be respectively performed considering the glenoid joint line and the scapular body axis in the axial plane.

### Indications

The clear indication for DAS lacks consensus. In biomechanical studies, when compared to Bankart alone, DAS seems to better restore anterior and anteroinferior glenohumeral translation in bone defects if smaller than 20%^17^^,^^18^ when labral repair is added.^19^ Nonetheless, with larger defects, the Latarjet procedure seems to outperform DAS. Therefore, for bone defect sizes that do not classically benefit from bone block procedures but for which isolated Bankart repair may be insufficient, DAS may be an alternative option, especially in the presence of concomitant SLAP lesions and when an engaging Hill-Sachs defect is absent.^11^^,^^12^

### Surgical Technique

**Fig 1:**
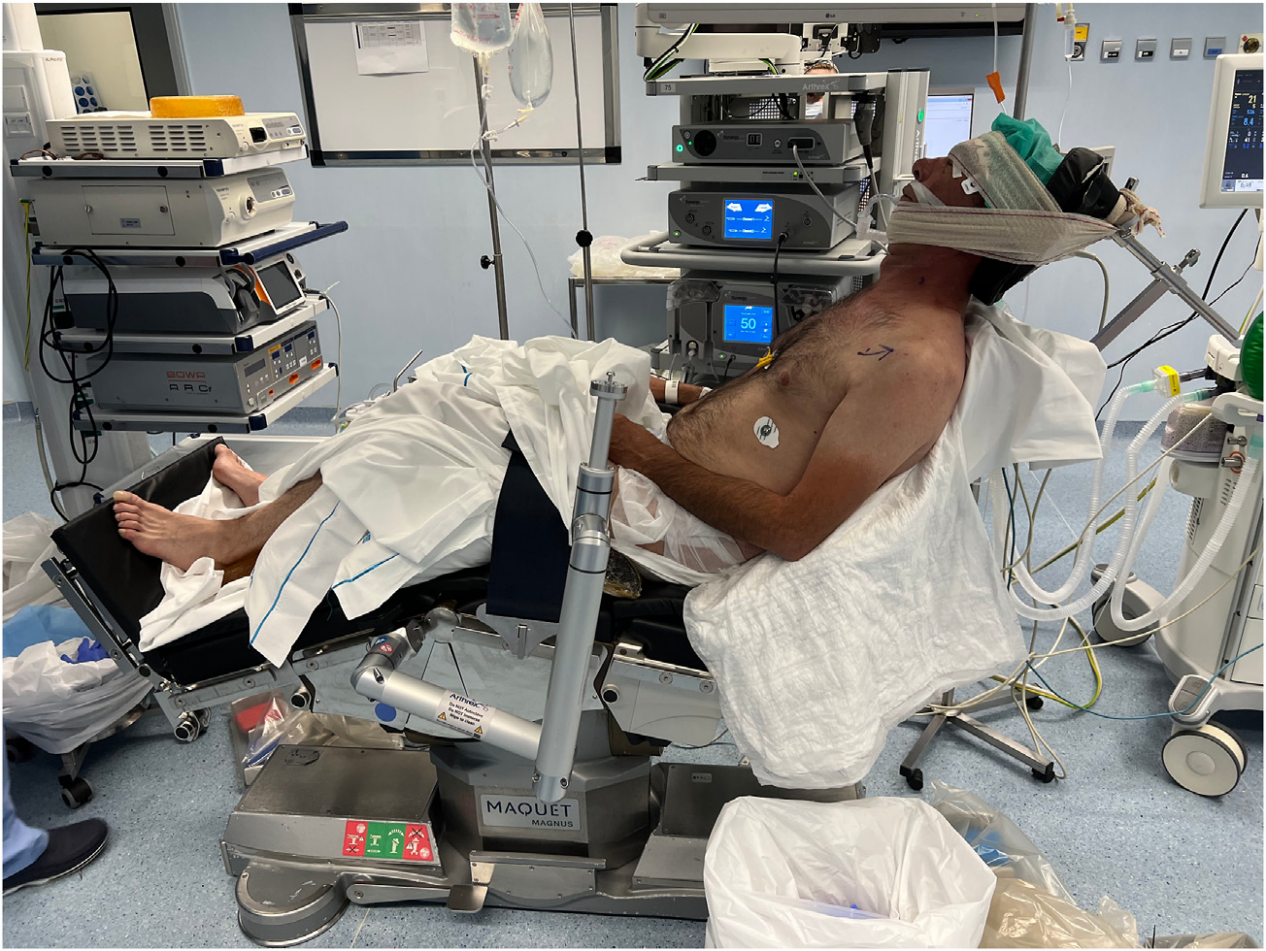
Patient position in beach chair.

After a combined anesthesia (brachial plexus neurologic block plus sedation), the patient is positioned in the beach-chair position (Fig 1).

**Fig 2:**
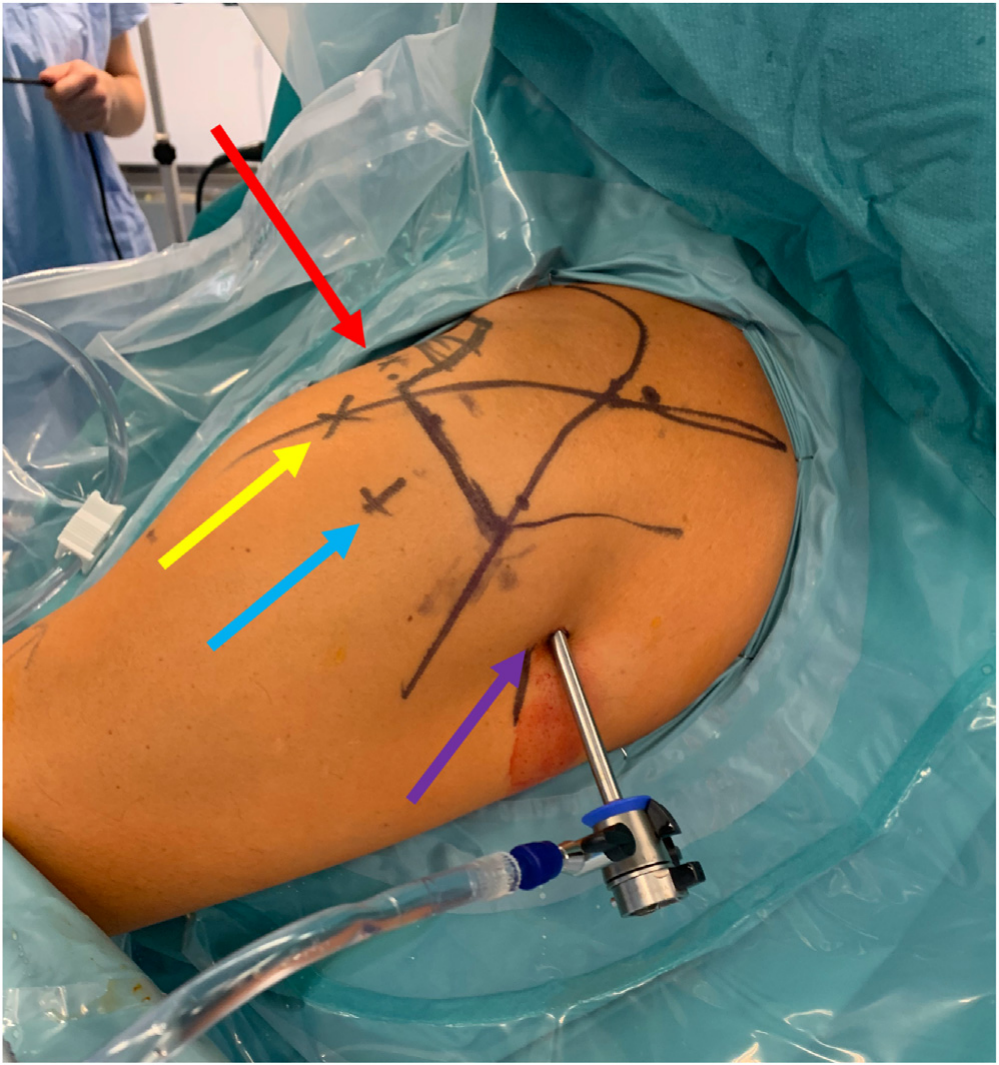
Left shoulder portals in beach-chair position: posterior portal (purple arrow) with the trocard sleeve inserted; lateral portal (blue arrow); anterosuperior portal (yellow arrow); anterosuperior medial portal (red arrow).

Cefazolin prophylaxis is administered, and the safety checklist is performed. After adequate disinfection and draping, anatomical references and portals are marked (Fig 2):

- Posterior (P)
- Anterosuperior (AS): 1 to 2 cm below the anterolateral acromion angle
- Anterosuperior medial (ASM): just lateral to the coracoid
- Lateral (L): 2 to 3 cm posterior to the AS portal
- Drill sleeves portal: created after the drill guide is inserted through the posterior portal and can vary according to the patient anatomy

Using a 16-gauge needle, joint inflation is performed using the posterior portal.

**Fig 3:**
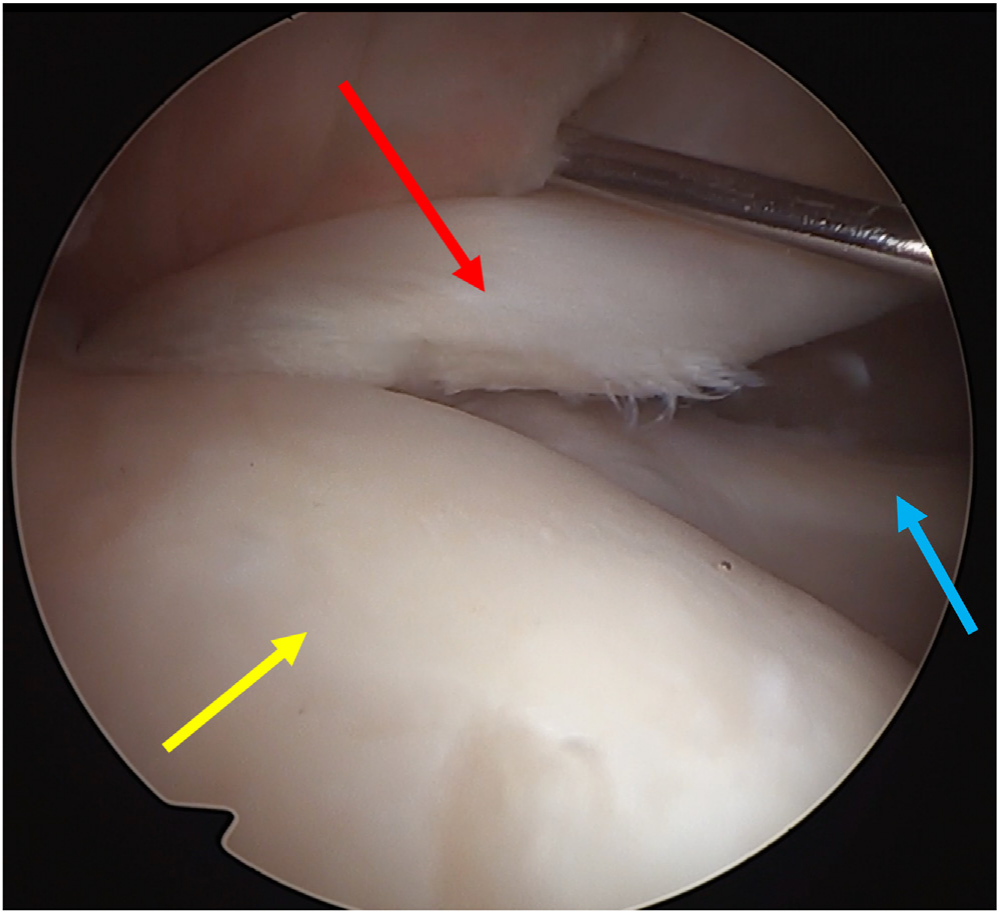
Biceps quality and excursion assessment (biceps: red arrow; humeral head: yellow arrow; subscapularis: blue arrow). Notice the slight fraying of the biceps tendon that, although it does not preclude the procedure, may increase the risk of subsequent tear (patient in beach-chair position; left shoulder).

The glenohumeral joint is inspected and all the expected and associated injuries are identified and described. Special care is given to biceps quality and excursion assessment (Fig 3).

**Fig 4:**
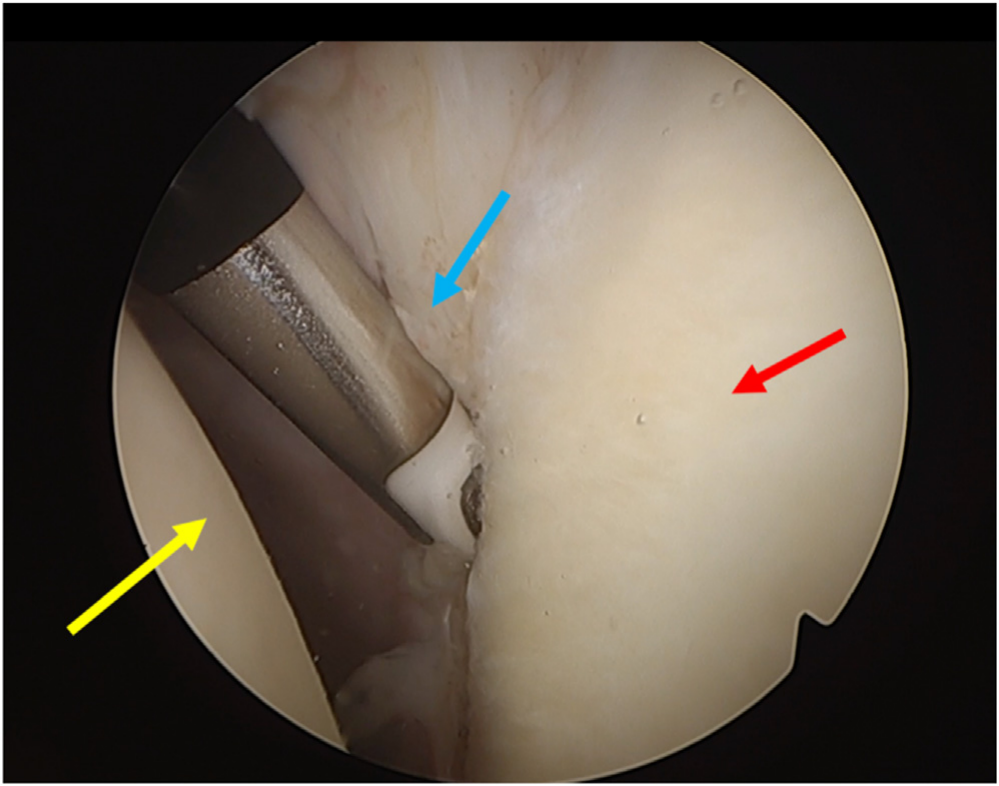
Labral detachment using a bisel-shaped radiofrequency device (glenoid: red arrow; humeral head yellow arrow; labrum: blue arrow) (patient in beach-chair position; left shoulder).

**Fig 5:**
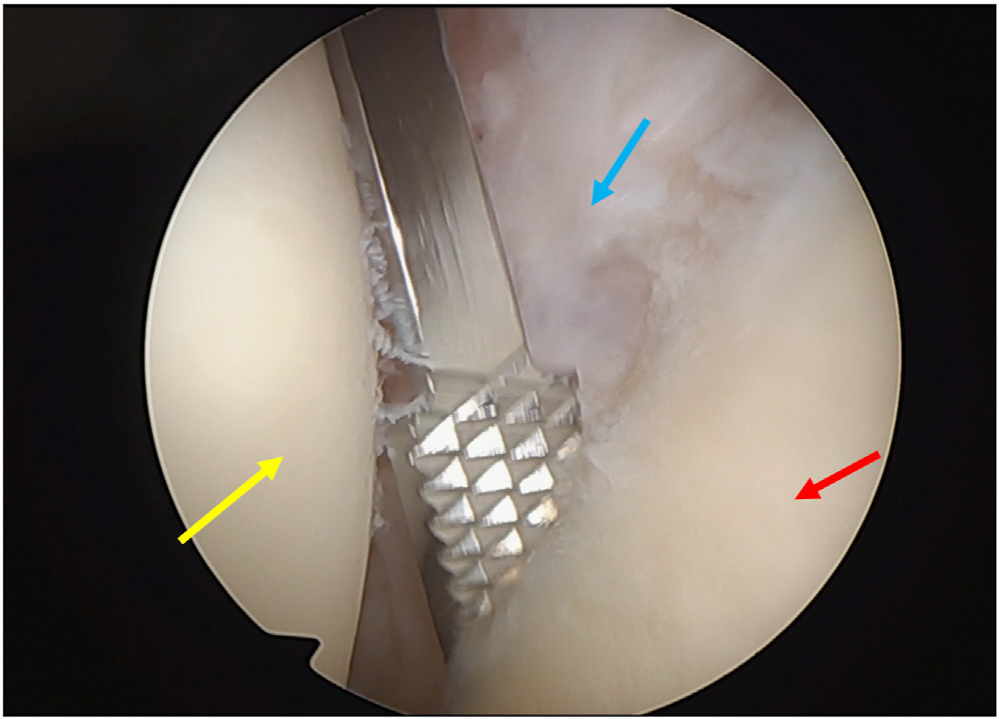
Labral detachment completion using a manual abrasive instrument (glenoid: red arrow; humeral head yellow arrow; labrum: blue arrow) (patient in beach-chair position; left shoulder).

The AS portal is created, and using a Werewolf radiofrequency (RF) device (Smith and Nephew), the labrum and capsule are detached (Fig 4). The anterior glenoid rim is then debrided with the soft tissue shaver (Smith and Nephew), and manual mechanical abrasion instruments (Fig 5) are used to create a bleeding bed.

The rotator interval is then opened and cleaned.

**Fig 6:**
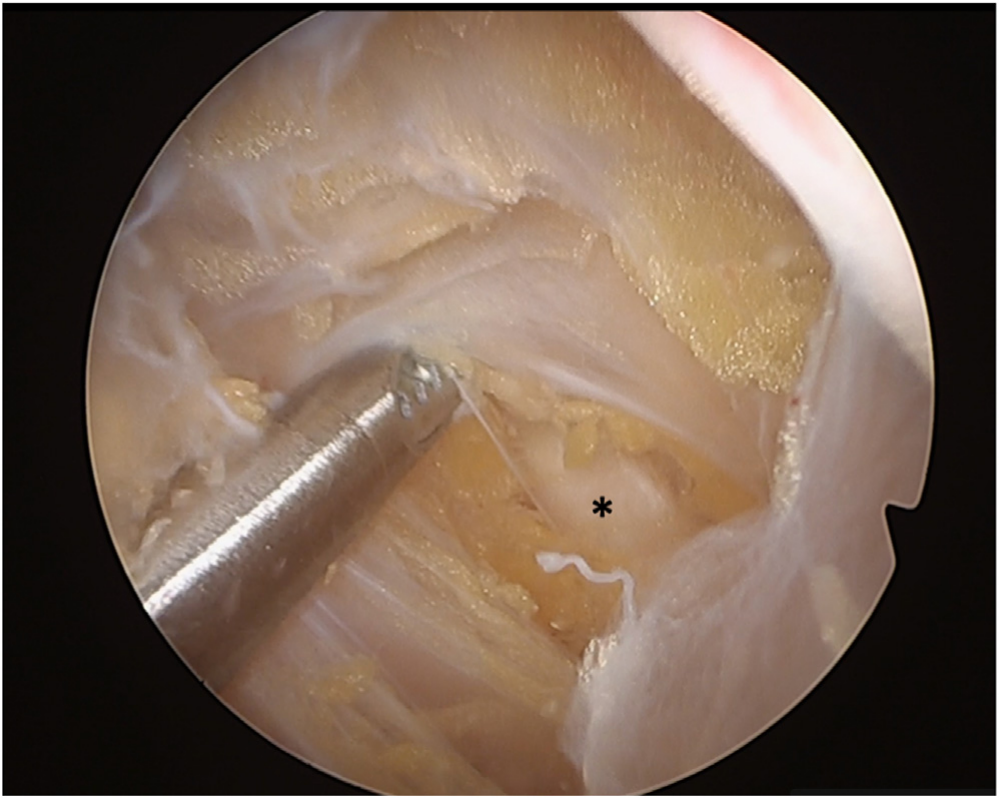
Axillary nerve (asterisk) (patient in beach-chair position; left shoulder; view from anterosuperior portal).

Care should be given to identifying the axillary nerve to prevent its injury (Fig 6 and Video 1). This is done by simple dissection using the shaver tip (with no motion or aspiration) or the switching stick (SST).

**Fig 7:**
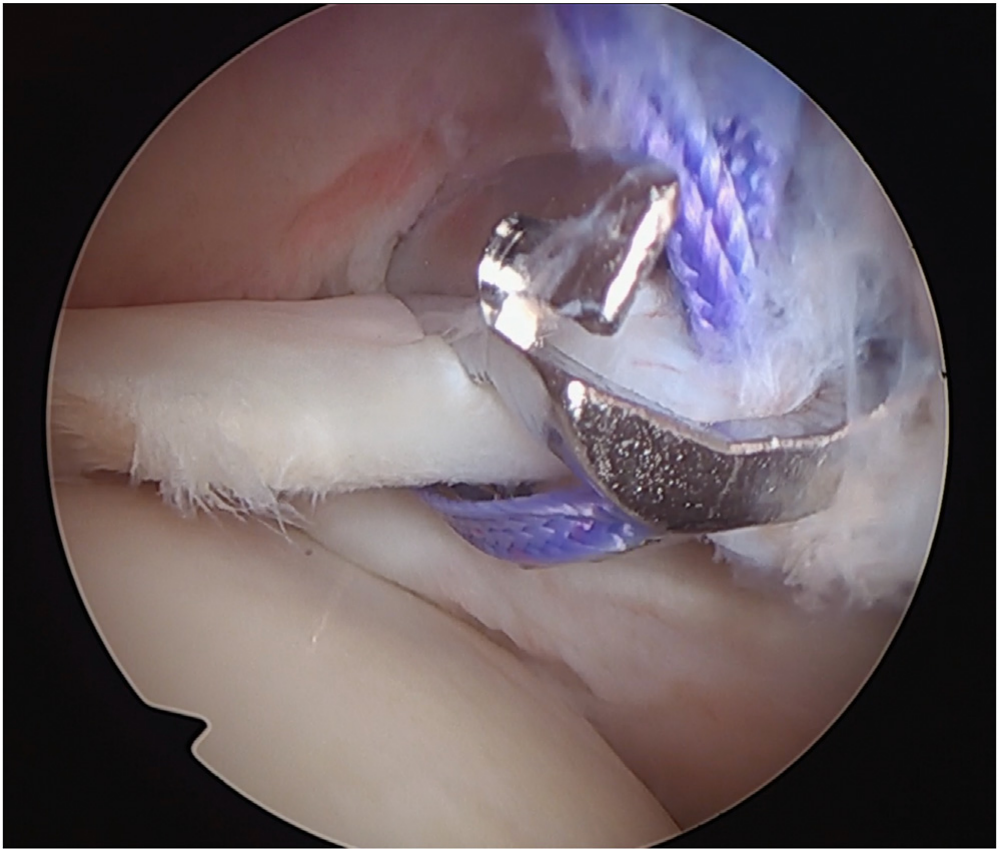
Biceps preparation using a mechanical suture passer (patient in beach-chair position; left shoulder).

**Fig 8:**
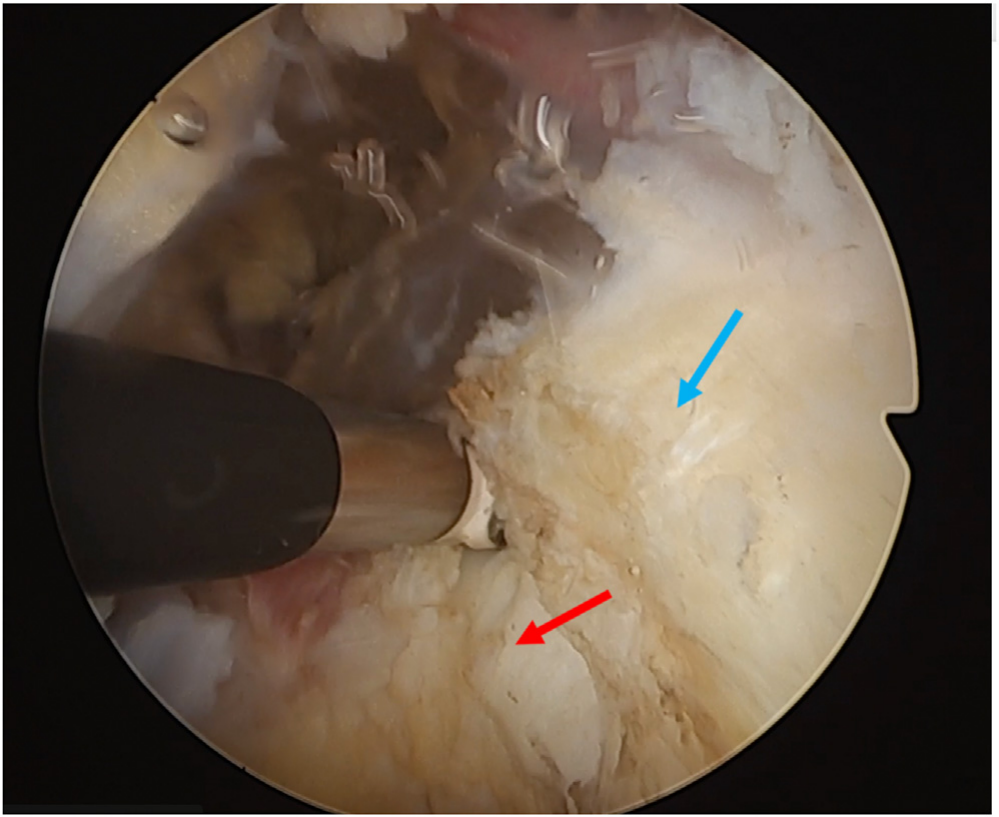
Subscapularis split execution: camera is in the anterosuperior portal and placed in the subcoracoid space (extra-articular). Radiofrequency device is placed in the anterosuperior medial portal. The superior third of the subscapularis is the blue arrow and the split is the red arrow (patient in beach-chair position; left shoulder).

**Fig 9:**
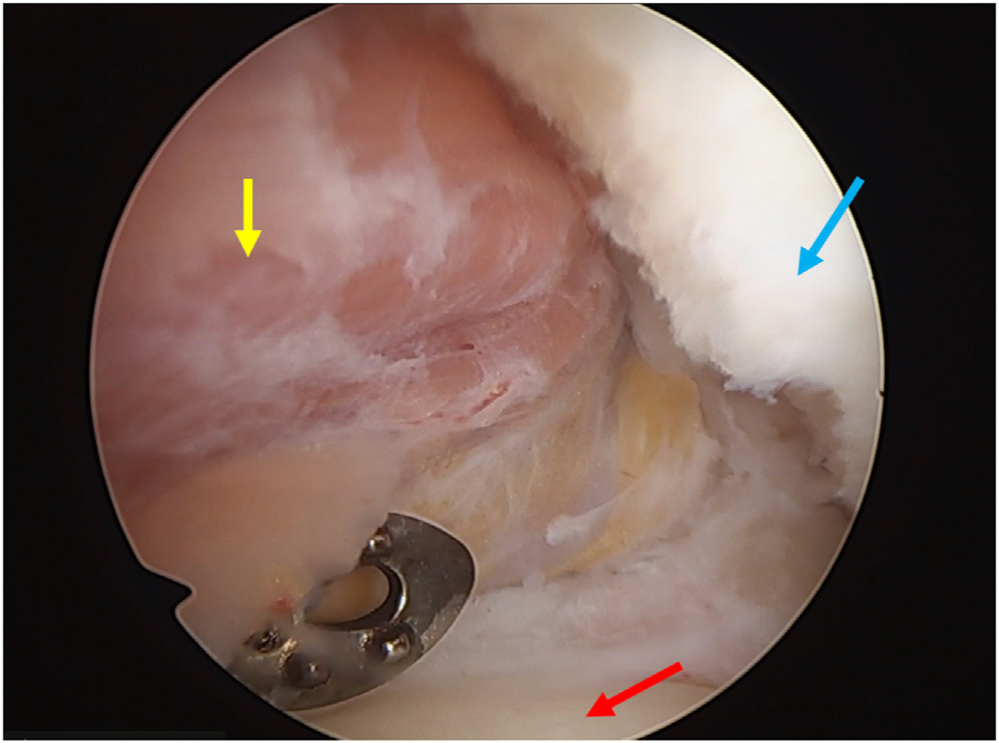
Subscapularis split seen from inside the joint. Camera is in the anterosuperior portal and radiofrequency device is placed in the split. Humeral head: red arrow; glenoid: blue arrow; superior third of the subscapularis: yellow arrow (patient in beach-chair position; left shoulder).

**Fig 10:**
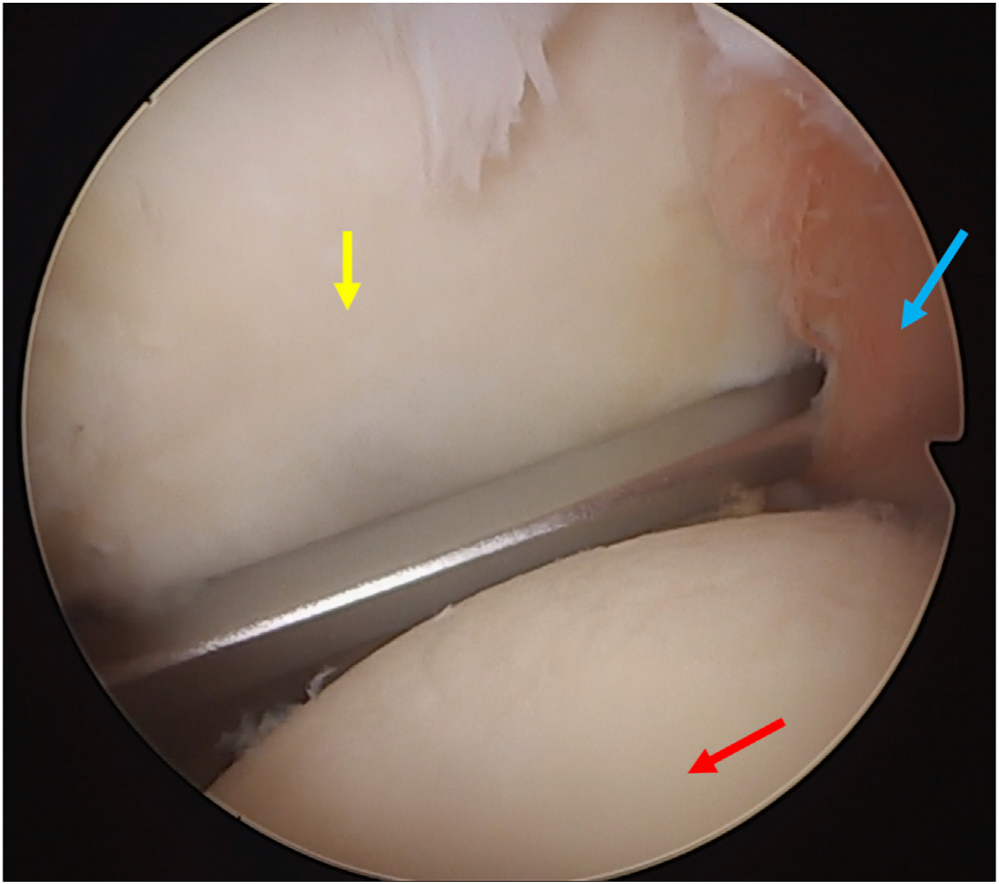
Bone tunnel drill guide. View from the anterosuperior portal (humeral head: red arrow; glenoid: yellow arrow; posterior capsule: blue arrow; patient in beach-chair position; left shoulder).

The biceps is then prepared by passing 2 different color high-resistance sutures lasso loops in the intra-articular portion of the LHB (Fig 7). This can be done with several different instruments, but we prefer to use a FirstPass mechanical suture passer (Smith and Nephew). The ASM portal is then created, and the camera is passed to the AS portal. Through the ASM portal, the necessary anterior and superior subacromial debridement is performed, followed by the subscapularis split (Fig 8). This is done using the Werewolf (Smith and Nephew) below the superior half of the tendon/muscle and with the camera in the AS portal controlling the procedure from inside the joint, posterior to the subscapularis, and outside, anterior to the subscapularis (Fig 9). The shaver should complete the split. This view is also ideal to control the insertion of the Latarjet Drill Guide (Smith and Nephew), that enters the joint though the posterior portal and is positioned at the level or slightly inferior to the glenoid “equator” level (Fig 10).

**Fig 11:**
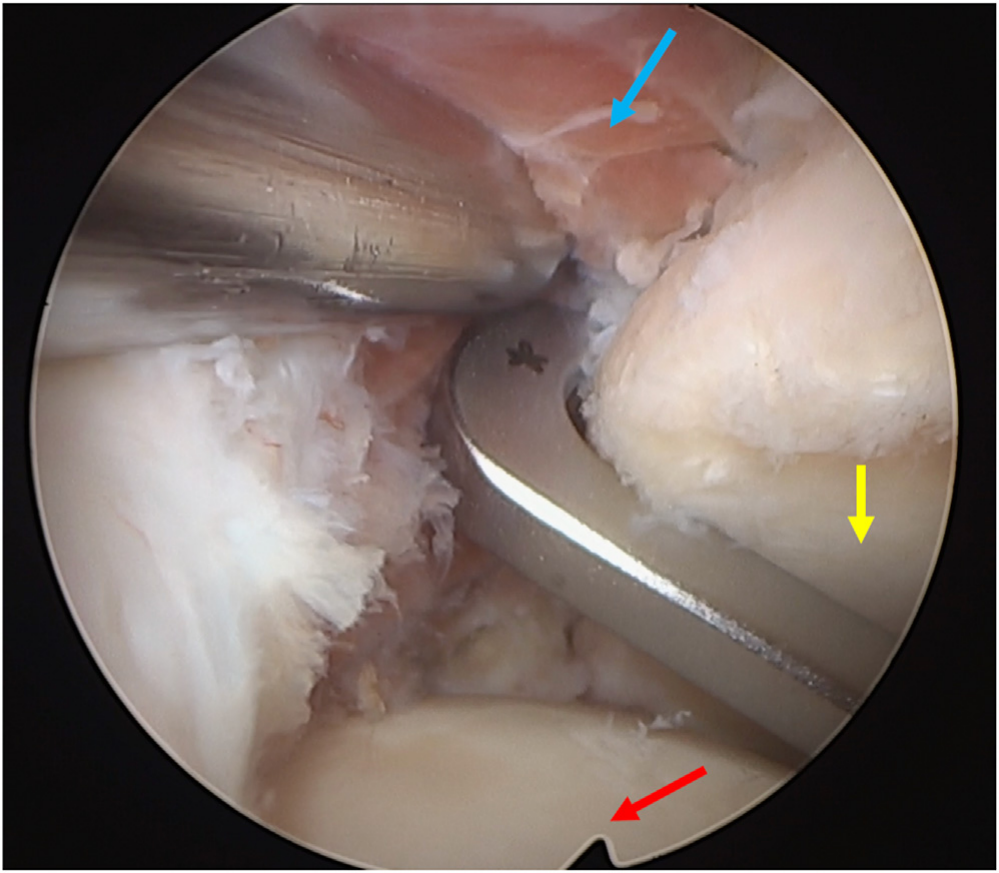
Bone tunnel drill guide applied in the anterior glenoid. View from the anterosuperior portal (humeral head: red arrow; glenoid: yellow arrow; subscapularis: blue arrow (patient in beach-chair position; left shoulder).

**Fig 12:**
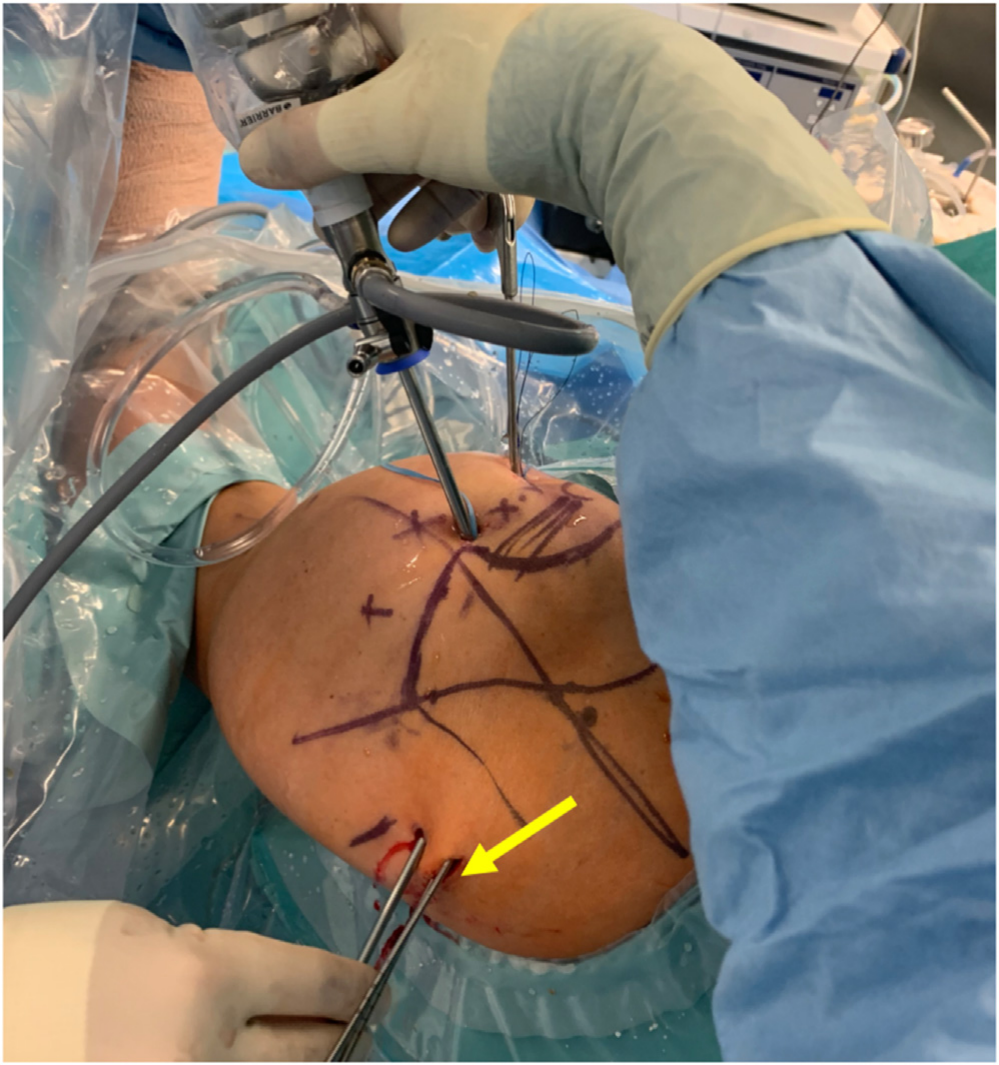
Patient is in the beach-chair position. Left shoulder external view of both tunnel drill guides applied from posterior to anterior (yellow arrow).

Having confirmed adequate position of the drill guide, the SST is placed in the ASM portal and retracts the subscapularis anteriorly, allowing an adequate view of the anterior glenoid (Fig 11). Both superior and inferior cannulated drill bits are inserted from posterior to anterior after an accessory single incision is created specifically for the drill sleeves, which are pushed blindly and directly into the posterior glenoid bone (Fig 12).

**Fig 13:**
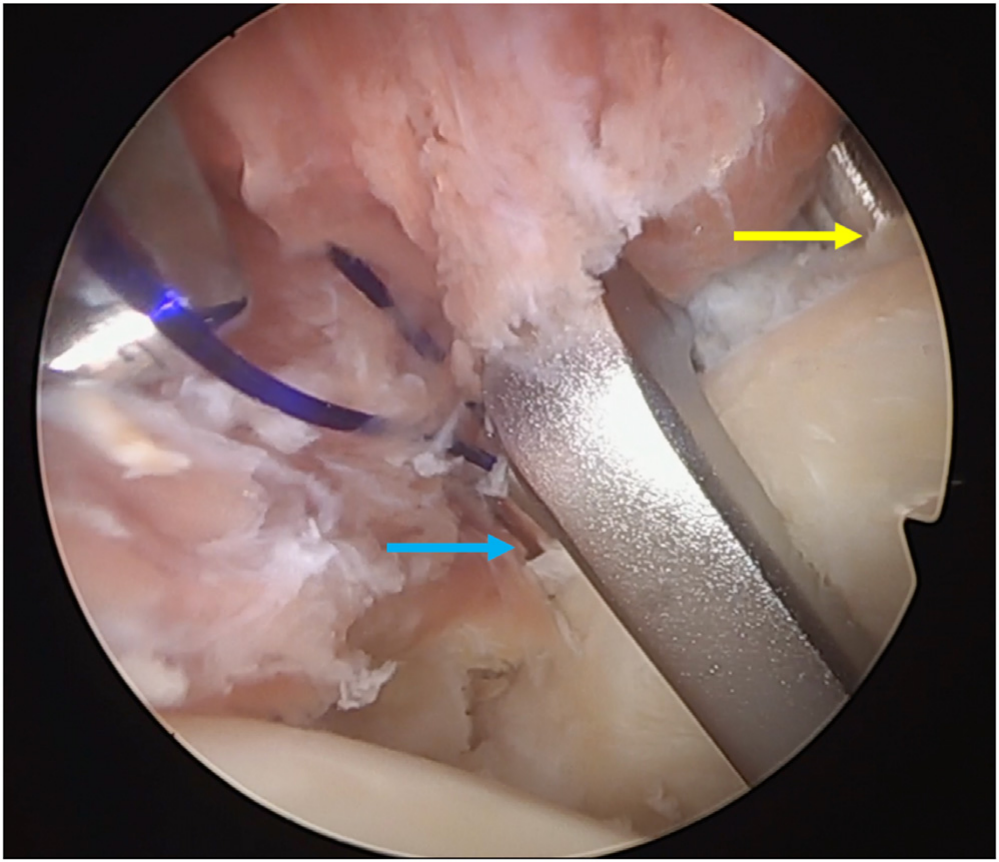
Both drill sleeves exiting from anterior glenoid (superior drill sleeve: yellow arrow; inferior drill sleeve: blue arrow). Left shoulder of a patient is in the beach-chair position with camera in the anterosuperior portal.

After removing the drill bit, its cannulated portion is left inside the glenoid, and 2 different color lasso shuttles are passed from posterior to anterior and retrieved outside at the ASM portal and through the subscapularis split (Fig 13).

**Fig 14:**
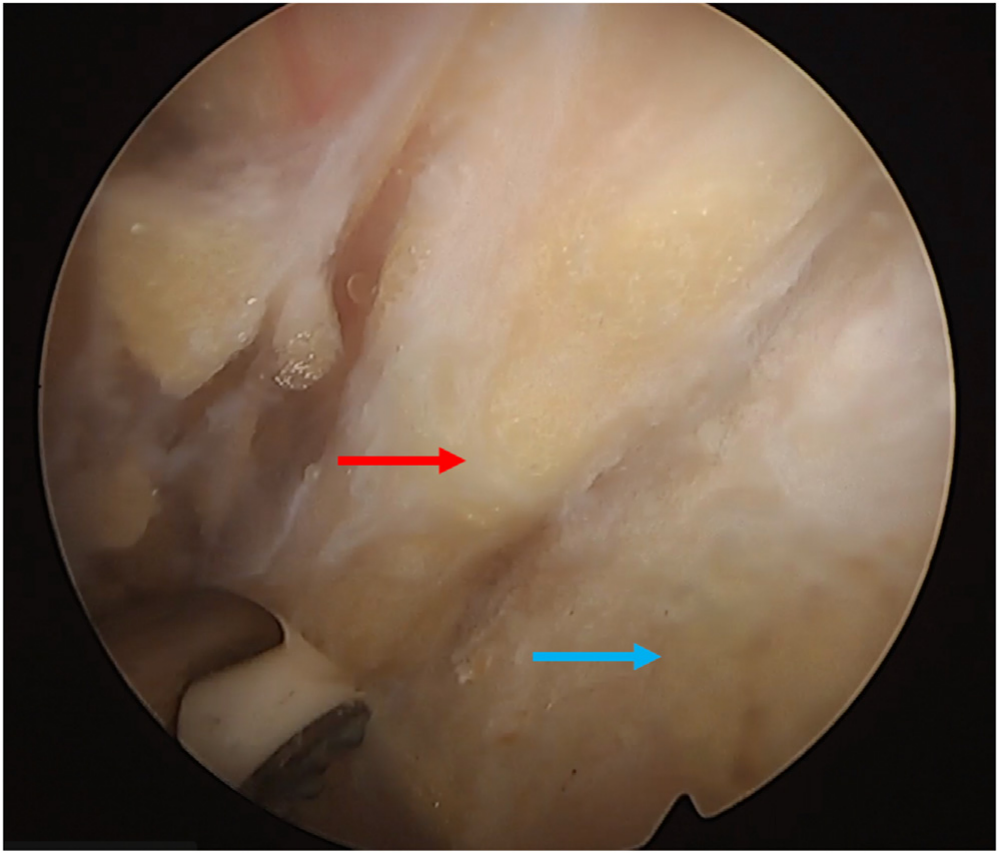
Extra-articular biceps gutter identification. Biceps synovial sheet (red arrow) and the lateral humeral cortex (blue arrow) are seen. Camera is in the lateral portal of a patient's left shoulder in the beach-chair position.

At this this time, one should prepare the extraction of the biceps outside of the joint, so the L portal may be useful to serve as a viewing portal, allowing the AS portal to be used as a working portal to open the biceps gutter using the Werewolf (Smith and Nephew) (Fig 14). To avoid LHB injury or its sectioning, the specific use of a beveled/angled tip, as the one mentioned, is helpful to progressively enter the gutter from posterior to anterior. When the gutter depression is felt, simple pressure creates the dissection plane in which to work.

After identifying the biceps extra-articularly, the surgeon re-enters the joint from the posterior portal or from the rotator interval window while performing the LHB tenotomy from the ASM portal.

The LHB is then pulled out of the joint without the need to release the superior gleno-umeral ligament or the transverse ligament.

**Fig 15:**
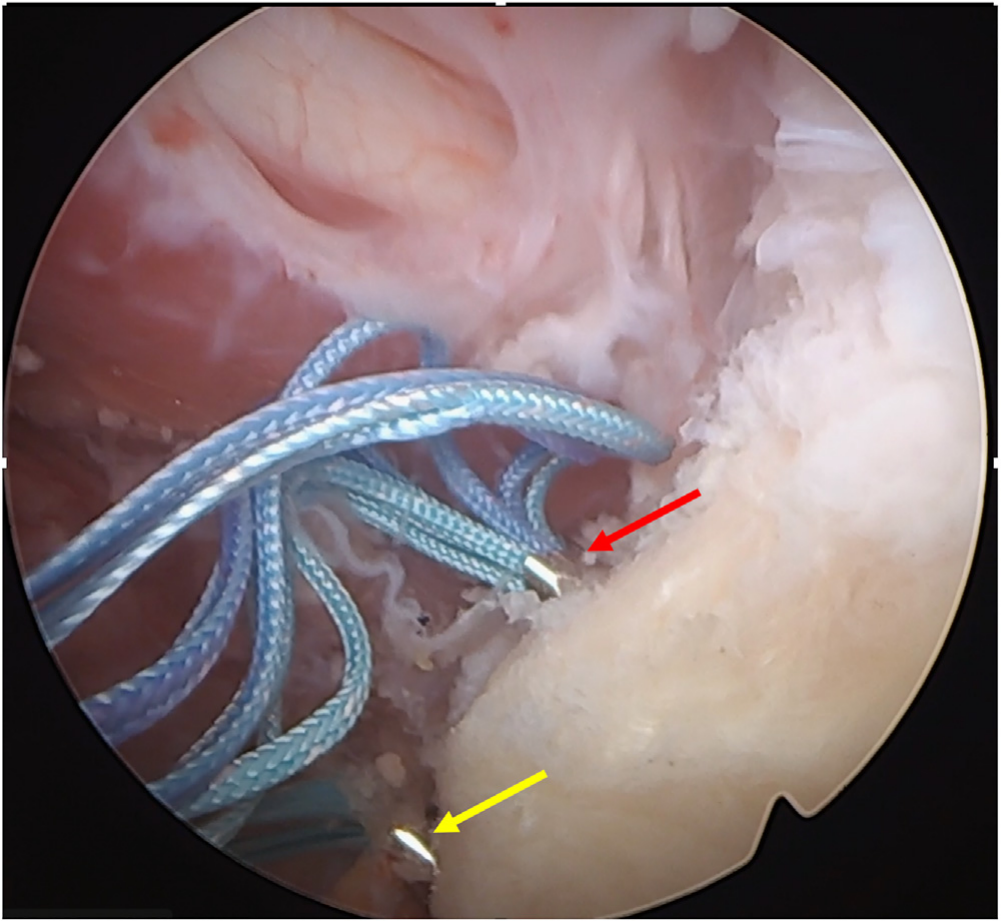
Both lasso loops of the extra-articularly prepared biceps tendon are passed through the subscapularis split after the previously applied suture shuttles were pulled back. Superior (red arrow) and inferior (yellow arrow) tunnel drill sleeves are seen with the suture wires passed in the long head of the biceps tendon entering them. (Patient in the beach-chair position; left shoulder; camera in the anterosuperior portal.)

At this stage, the biceps is pulled out through the AS portal and lasso loop quality is verified and, if needed, a third reinforcing lasso loop can be added. After reintroduction of the biceps into the subacromial space, the surgeon pulls both the inferior LHB lasso loops and the inferior tunnel shuttle loop through the same portal (normally the ASM), and the lasso is shuttled into the inferior tunnel with direct arthroscopic control of the passage. The same gesture is performed for the superior lassos and superior tunnel shuttle (Fig 15). While performing this gesture, placing the camera in the L portal can be helpful to allow an SST to be applied as a soft tissue spreader allowing an adequate visualization of suture passage in the subscapularis split.

**Fig 16:**
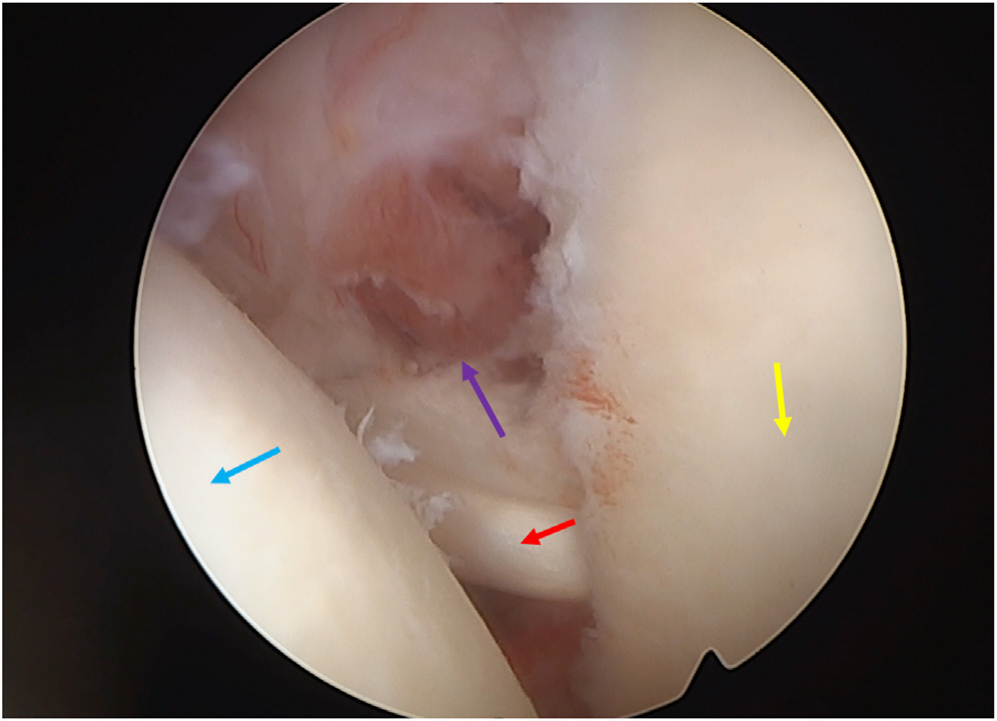
Final construct in left shoulder of a patient in the beach-chair position: the camera is in the posterior portal. On the left side, the humeral head can be seen (blue arrow), while on the right, one can observe the glenoid (yellow arrow). In the center part of the image, the long head of the biceps (red arrow) can be seen serving as an anterior sling while the subscapularis can be identified above (purple arrow).

Sutures are then pulled in the back while the shoulder and the transferred biceps stability are tested. Four to 5 half hitch knots are then sequentially tied over both superior and inferior mini round endobuttons (Smith and Nephew) (see Video 1) while the arm is at 45° of forward flexion and 30° of external rotation, with the elbow fully extended. The final repair is then evaluated from the posterior portal (Fig 16).

Portals are then closed, and the patient is placed in an arm sling for 4 weeks, allowing only passive elbow flexion and extension movements, while no shoulder movement is allowed (except for bathing and dressing).

A stepwise approach is shown in Table 1.

**Table 1:** Stepwise Approach to the Described Technique

Step	Surgical Procedure
1.	Joint inspection
2.	Assess labral and biceps quality
3.	Detach the labrum
4.	Prepare the glenoid rim
5.	Open the rotator interval
6.	Find the axillary nerve
7.	Prepare the biceps with 2 lasso loops
8.	In the subacromial space, prepare the anterior region
9.	Do the subscapularis split
10.	Introduce the bone tunnel guide from the posterior portal
11.	Tunnel drilling while retracting the subscapularis anteriorly
12.	Pass 2 different color sutures in the drill sleeves of the bone tunnels and retrieve them through the subscapularis split
13.	Tenotomize and extract the LHB to the subacromial space by opening the transverse ligament
14.	Pass the LHB sutures in a correspondent manner into the bone tunnels (superior LHB suture to the superior bone tunnel and inferior to the inferior one)
15.	Pull the LHB into the joint
16.	Fix the tendon by applying to mini round endobuttons posteriorly with the arm at 30° of external rotation
17.	Confirm adequate release and excursion of the LHB in the gutter and release it distally if necessary

LHB, long head of the biceps.

The patient is then referred to physical therapy and hydrotherapy to regain range of motion for the next 4 to 8 weeks (8-12 postoperative weeks), while initiating scapular strengthening protecting both the biceps and the repair. At the eighth postoperative week, the patient starts performing biceps isometric strengthening exercises and active range of motion.

At the 12th postoperative week, full range of motion exercises are allowed. The patient is only allowed to start noncontact sport training at the 20th postoperative week, while in case of contact/high risk of fall sports, this is only allowed at the 24th postoperative week.

## Discussion

We describe an onlay, anchorless, and intra-articular knotless method of fixing the LHB into the anterior glenoid that provides the important stabilizing “sling effect” responsible for the low rates of redislocation reported in studies using the Latarjet procedure.^20^^,^^21^ Our technique also allows the addition of an inferior capsular plication if the tissue is of good quality, by simply passing a suture in the inferior capsule and passing it in the inferior tunnel.

The largest and the longest follow-up retrospective case series published on DAS clinical outcomes reported an unexpectedly high incidence of recurrence at a minimum follow-up of 2 years. The authors of the study justified the 13.6% recurrence rate with inadequate LHB tendon tension and/or LHB fixation,^22^ related not only to the method of fixation of the LHB (inlay fixation) but to the type implants used as well.

Other inlay DAS techniques have been described^11^^,^^23^^,^^24^ using button devices, but they do not use any type of specific guide. Therefore, they pose an increased risk of iatrogenic suprascapular nerve injury due to inadequate tunnel placement, as Garcia et al.^23^ recognized. This risk has also been described and studied for the Latarjet procedure.^25^, ^26^, ^27^ Also, the use of drilling instruments anteriorly, especially through the subscapularis split, increases the risk of iatrogenic neurologic injury due to the proximity of both the axillary and the musculocutaneous nerves.^28^, ^29^, ^30^

Alternative onlay techniques were developed as well, and one of the purposes was to avoid the potential pitfalls of inlay DAS.^13^^,^^15^^,^^31^ Some onlay DAS techniques allow for multiple fixation points in the anterior glenoid, increasing both the rotational stability of the transferred LHB and its healing potential.^32^ Another reported advantage of some onlay techniques is the labroplasty effect,^13^^,^^14^^,^^31^ although the stabilizing value of that isolated gesture is not fully supported by the literature.^33^

Each of the previously described onlay techniques relies on a traction-countertraction mechanism, to deliver or fix the LHB tendon into the glenoid, regardless if double pulley^31^ or suture pulling in knotless anchors^13^^,^^15^ mechanisms are used. These delivery or fixation methods, although theoretically sufficient and robust to fix the labrum and other noncontractile tissues, may be subjected to higher degrees of stress when used to pull a contractile tissue that generates force in the opposite direction, as is the case of the LHB. Moreover, in the technique by Azevedo and Ângelo,^31^ we also found that using multiple strands of suture in a double-pulley mechanism in the anterior region of the shoulder created a high risk of suture tangling during suture passage and knot tying, which increases the risk of complications and surgical time, while also enlarging the technique learning curve as high-level expertise in suture management is required. Our technique addresses these pitfalls and, while not producing the labroplasty effect, does permit an inferior capsuloplasty if the tissue is adequate.

As shown in Tables 2 and 3, our technique has several theoretical advantages. We use 2 small bone tunnels, which are almost half the diameter of the smallest inlay tunnels used in other techniques (2.8 mm vs 4.5 mm); therefore, more glenoid bone stock is preserved. We avoid the use of anchors in the anterior glenoid surface; therefore, the risk of intra-articular implant-related complications is reduced.

**Table 2:** Dynamic Anterior Stabilization Technical Variants Comparison

	Collin et al.^22^	Garcia et al.^23^	Nair et al.^24^	Tang and Zhao^11^	Milenin and Toussaint^13^	Popescu et al.^15^	Azevedo et al.^31^	Maia Dias
Type of DAS	Inlay	Inlay	Inlay	Inlay	Onlay	Onlay	Onlay	Onlay
Fixation mechanism	Single interference screw	Single adjustable loop button	Single Adjustable loop button	Single Adjustable loop button	Knotless anchors	Knotless anchors	Tied suture anchors	Two mini-round endobuttons
Fixation mechanism instrumentation	Anterior—trans SSC split (intra-articular)	Anterior—trans SSC split (intra-articular)	Anterior—trans SSC split (intra-articular)	Posterior extra-articular	Anterior—rotator interval (intra-articular)	Anterior—rotator interval (intra-articular)	Anterior—trans SSC split (intra-articular)	Posterior—extra-articular
Guided instrumentation?	No	No	No	No	No	No	No	Yes
Risks	• Inadequate tendon tension • Bone tunnel fracture • Bone tunnel resorption • Suprascapular nerve injury • Anterior neurologic injury	• Inadequate tendon tension • Bone tunnel fracture • bone tunnel resorption • Suprascapular nerve injury • Anterior neurological injury • Hardware related—artifacts in future MR imaging	• Inadequate tendon tension • Bone tunnel fracture • Bone tunnel resorption • Suprascapular nerve injury • Anterior neurologic injury • Hardware related—artifacts in future MR imaging	• Inadequate tendon tension • Bone tunnel fracture • Bone tunnel resorption • Suprascapular nerve injury • Hardware related—artifacts in future MR imaging	• Anterior neurologic injury • Anchor loosening/fracture • Pull-counterpull mechanism • Anterior bone loss • Difficult suture management • Knot-tying failure	• Anterior neurologic injury • Anchor loosening/fracture • Pull-counterpull mechanism • Anterior bone loss • Risk of failure of the only 1 point of fixation of the LHB	• Anterior neurologic injury • Counterpull mechanism • Anchor loosening/fracture • Anterior bone loss • Difficult suture management • Knot-tying failure	• Knot-tying failure • Excessive LHB medialization • Hardware-related—artifacts in future MR imaging • Bone tunnel fracture

DAS, dynamic anterior shoulder stabilization; LHB, long head of the biceps; MR, magnetic resonance; SSC, subscapularis.

**Table 3:** Advantages and Disadvantages of the Current Technique

Advantages	Disadvantages
Guided instrumentation	Medialization of the LHB transfer with loss of labroplasty effect
Several LHB fixation points	Multiple LHB kinking points
No rigid material in the joint or in the bone	Knot-tying problems
No anterior drilling/risky instrumentation	Metal buttons create artifacts in postoperative MRI

LHB, long head of the biceps; MRI, magnetic resonance imaging.

Our technique may also minimize the risk of iatrogenic neurologic injury because no drilling instruments are placed anteriorly to fix the LHB, and we use a specific guide that allows the small round buttons to be placed in the posterior “glenoid safe zone,“^30^^,^^32^ thus reducing the risk of suprascapular nerve injury.

However, some pitfalls (Table 3) related to the onlay technique are not completely avoided in the current technique. First, the use of multiple location fixation points, albeit allowing for a more robust fixation, also generates 1 additional kinking point in the LHB path. This can increase the risk of failure^34^ due to excessive stiffness of the fixation and increased stress at the most inferior fixation point.^34^, ^35^, ^36^

Second, in the current technique, the transfer is medialized by 6 mm, which can theoretically increase the risk of recurrence and reduces the labroplasty effect, and because metal buttons are used, postoperative MRI to assess LHB healing may be harder to assess.

In conclusion, this guided anchorless technique may simplify and make the most difficult and critical steps of DAS safer and reproducible.

## Disclosure

The authors report no conflicts of interest in the authorship and publication of this article. Full ICMJE author disclosure forms are available for this article online, as supplementary material.

## Supplementary Data

ICMJE author disclosure forms

Video 1 Video demonstrating an onlay, anchorless, and intra-articular knotless dynamic anterior shoulder stabilization procedure performed in a left shoulder of a 43-year-old man.

## References

[1] Shin S.J., Kim R.G., Jeon Y.S., Kwon T.H. (2017). Critical value of anterior glenoid bone loss that leads to recurrent glenohumeral instability after arthroscopic bankart repair. Am J Sports Med.

[2] Shaha J.S., Cook J.B., Song D.J. (2015). Redefining ‘critical’ bone loss in shoulder instability. Am J Sports Med.

[3] Ahmed I., Ashton F., Robinson C.M. (2012). Arthroscopic Bankart repair and capsular shift for recurrent anterior shoulder instability: Functional outcomes and identification of risk factors for recurrence. J Bone Jt Surg.

[4] Haroun H.K., Sobhy M.H., Abdelrahman A.A. (2020). Arthroscopic Bankart repair with remplissage versus Latarjet procedure for management of engaging Hill-Sachs lesions with subcritical glenoid bone loss in traumatic anterior shoulder instability: A systematic review and meta-analysis. J Shoulder Elb Surg.

[5] Hurley E.T., Toale J.P., Davey M.S. (2020). Remplissage for anterior shoulder instability with Hill-Sachs lesions: A systematic review and meta-analysis. J Shoulder Elb Surg.

[6] Sano H., Komatsuda T., Abe H., Ozawa H., Yokobori T.A. (2020). Proximal-medial part in the coracoid graft demonstrates the most evident stress shielding following the Latarjet procedure: A simulation study using the 3-dimensional finite element method. J Shoulder Elb Surg.

[7] Carbone S., Moroder P., Runer A., Resch H., Gumina S., Hertel R. (2016). Scapular dyskinesis after Latarjet procedure. J Shoulder Elb Surg.

[8] Cerciello S., Corona K., Morris B.J., Santagada D.A., Maccauro G. (2019). Early outcomes and perioperative complications of the arthroscopic Latarjet procedure: Systematic review and meta-analysis. Am J Sports Med.

[9] Hurley E.T., Schwartz L.B., Mojica E.S. (2021). Short-term complications of the Latarjet procedure: A systematic review. J Shoulder Elb Surg.

[10] Imam M.A., Shehata M.S., Meyer D.C. (2020). Bankart repair versus Latarjet procedure for recurrent anterior shoulder instability: A systematic review and meta-analysis of 3275 shoulders. Am J Sports Med.

[11] Tang J., Zhao J. (2017). Arthroscopic transfer of the long head of the biceps brachii for anterior shoulder instability. Arthrosc Tech.

[12] Collin P., Lädermann A. (2018). Dynamic anterior stabilization using the long head of the biceps for anteroinferior glenohumeral instability. Arthrosc Tech.

[13] Milenin O., Toussaint B. (2019). Labral repair augmentation by labroplasty and simultaneous trans-subscapular transposition of the long head of the biceps. Arthrosc Tech.

[14] de Campos Azevedo C., Ângelo A.C. (2023). Onlay dynamic anterior stabilization with biceps transfer for the treatment of anterior glenohumeral instability produces good clinical outcomes and successful healing at a minimum 1 year of follow-up. Arthrosc Sport Med Rehabil.

[15] Popescu I.A., Neculau D.C., Simion C., Popescu P. (2022). Modified dynamic anterior stabilization (DAS) and Hill-Sachs remplissage for the treatment of recurrent anterior shoulder dislocation. Arthrosc Tech.

[16] Beighton P., Solomon L., Soskolne C.L. (1973). Articular mobility in an African population. Ann Rheum Dis.

[17] Lobao M.H., Abbasi P., Murthi A.M. (2022). Long head of biceps transfer to augment Bankart repair in chronic anterior shoulder instability with and without subcritical bone loss: A biomechanical study. J Shoulder Elb Surg.

[18] Mehl J., Otto A., Imhoff F.B. (2019). Dynamic anterior shoulder stabilization with the long head of the biceps tendon: A biomechanical study. Am J Sports Med.

[19] Nicholson A.D., Carey E.G., Mathew J.I. (2022). Biomechanical analysis of anterior stability after 15% glenoid bone loss: Comparison of Bankart repair, dynamic anterior stabilization, dynamic anterior stabilization with Bankart repair, and Latarjet. J Shoulder Elb Surg.

[20] Giles J.W., Boons H.W., Elkinson I. (2013). Does the dynamic sling effect of the Latarjet procedure improve shoulder stability? A biomechanical evaluation. J Shoulder Elb Surg.

[21] Yamamoto N., Muraki T., An K. (2013). The stabilizing mechanism of the Latarjet procedure: A cadaveric study. J Bone Jt Surg.

[22] Collin P., Nabergoj M., Denard P.J., Wang S., Bothorel H., Lädermann A. (2022). Arthroscopic biceps transfer to the glenoid with Bankart repair grants satisfactory 2-year results for recurrent anteroinferior glenohumeral instability in subcritical bone loss. Arthroscopy.

[23] Garcia J.C., Mendes R.B., Muzy P.C., de Paiva Raffaelli M., Dumans e Mello M.B. (2023). Dynamic anterior stabilization of the shoulder with adjustable-loop device. Arthrosc Tech.

[24] Nair A.V., Mohan P.K., Jangale A. (2022). Dynamic anterior stabilization using transosseous bone tunnel technique with the adjustable loop length cortical button incorporating high-strength suture augmentation for recurrent shoulder instability. Arthrosc Tech.

[25] Sastre S., Peidro L., Méndez A., Calvo E. (2016). Suprascapular nerve palsy after arthroscopic Latarjet procedure: A case report and review of literature. Knee Surg Sport Traumatol Arthrosc.

[26] Longo U.G., Forriol F., Loppini M. (2015). The safe zone for avoiding suprascapular nerve injury in bone block procedures for shoulder instability. A cadaveric study. Knee Surg Sport Traumatol Arthrosc.

[27] Lädermann A., Denard P.J., Burkhart S.S. (2012). Injury of the suprascapular nerve during Latarjet procedure: An anatomic study. Arthroscopy.

[28] Delaney R.A., Freehill M.T., Janfaza D.R., Vlassakov K.V., Higgins L.D., Warner J.J.P. (2014). 2014 Neer Award Paper: Neuromonitoring the Latarjet procedure. J Shoulder Elb Surg.

[29] Boileau P., Mercier N., Old J. (2010). Arthroscopic Bankart-Bristow-Latarjet (2B3) procedure: How to do it and tricks to make it easier and safe. Orthop Clin North Am.

[30] Boileau P., Gendre P., Baba M. (2016). A guided surgical approach and novel fixation method for arthroscopic Latarjet. J Shoulder Elb Surg.

[31] De Campos Azevedo C., Angelo A.C. (2021). All-suture anchor dynamic anterior stabilization produced successful healing of the biceps tendon. JBJS Case Connect.

[32] Valenti P., Maroun C., Wagner E., Werthel J.D. (2018). Arthroscopic Latarjet procedure combined with Bankart repair: A technique using 2 cortical buttons and specific glenoid and coracoid guides. Arthrosc Tech.

[33] Wilk K.E., Arrigo C.A., Andrews J.R. (1997). Current concepts: The stabilizing structures of the glenohumeral joint. J Orthop Sports Phys Ther.

[34] Aida H.F., Shi B.Y., Huish E.G., McFarland E.G., Srikumaran U. (2020). Are implant choice and surgical approach associated with biceps tenodesis construct strength? A systematic review and meta-regression. Am J Sports Med.

[35] De Benedetto M., Galasso O. (2020). Arthroscopic Latarjet procedure: A technique using double round ENDOBUTTONs and specific glenoid and coracoid guides. Arthrosc Tech.

[36] Dias M., Gonçalves S., Completo A. (2022). Mechanical consequences at the tendon-bone interface of different medial row knotless configurations and lateral row tension in a simulated rotator cuff repair. J Exp Orthop.

